# Editorial: Plant secondary metabolites in food: absorption, metabolism and effects on glucolipid metabolism

**DOI:** 10.3389/fnut.2024.1382677

**Published:** 2024-04-17

**Authors:** Qing-Feng Zhang, Wen-Jun Wang, Shengbao Cai, Guo-Dong Zheng

**Affiliations:** ^1^Jiangxi Key Laboratory of Natural Product and Functional Food, College of Food Science and Engineering, Jiangxi Agricultural University, Nanchang, China; ^2^Faculty of Food Science and Engineering, Kunming University of Science and Technology, Kunming, China

**Keywords:** secondary metabolites, metabolism, absorption, glucolipid metabolism, phytochemicals

Plant foods are a major part of the human diet. Besides major nutrients of protein and carbohydrate, plant foods also contain many secondary metabolites, such as polyphenols, saponin, terpenoid, etc. Although these phytochemicals are the minor components in food, they exhibit very important roles in foods nutrition and physiological function. Epidemiological studies have shown that high consumption of fruits and vegetables is negatively associated with many chronic diseases, such as obesity, diabetes and cardiovascular disease (1). Besides dietary fibers, phytochemicals are mainly responsible for these effects because modern studies revealed that they exhibit the bioactivities of anti-oxidative, anti-microbial, anti-tumor, hepatoprotective, anti-inflammatory activities, etc. (2). However, many phytochemicals, particularly polyphenols, suffer from the shortcoming of poor bioavailability *in vivo* because of instability, low solubility and/or permeability (3). Usually, absorption is the prerequisite of phytochemicals to exert most bioactivities *in vivo*. Hence, the strategy to improve their bioavailability is a hot topic in nutrition study (4). Nowadays, the incidence of diabetes, non-alcoholic fatty liver disease (NAFLD), obesity and other disorders of glycolipid metabolism is on the rise, which has become a serious threat to people's health (5). Epidemiological studies have confirmed the positive effects of plant food on such chronic diseases. However, deeply revealing the regulation effects and mechanism of phytochemicals on glycolipid metabolism is in demand, particularly, based on their bioavailability and metabolism pattern *in vivo*.

This Research Topic is aimed at collecting studies about the absorption and metabolism of plant secondary metabolites as well as their effects on glucolipid metabolism. In this Research Topic, there are five papers covering the above-mentioned aspects.

In the study of Liu et al., the glucotoxicity protecting effects of honokiol, a natural biphenolic component derived from the dietary supplement Magnolia ocinalis extract, were investigated. Honokiol significantly increased glucose consumption and promoted GLUT2 translocation to the plasma membrane in glucosamine-treated HepG2 cells. Furthermore, honokiol alleviated glucotoxicity-induced oxidative stress by reducing ROS accumulation and loss of mitochondrial membrane potential. The actions of honokiol are AMPK activation-dependent. These findings provided new insights into the antidiabetic effects of honokiol.

Resinacein S is the major triterpenoids of *Ganoderma lucidum*. In the study of Mao et al., the protective effects of Resinacein S against NAFLD in high fat diet fed mice were investigated. Resinacein S can significantly change the lipid metabolism in liver cells and yield a protective effect against steatosis and liver injury. Protein–protein interactions network and RNA-seq analysis were used to characterize the targets of Resinacein S against NAFLD.

The gut microbiota is a vast and intricate microecosystem that play a crucial role in preserving the host's health with contributing to xenobiotic, nutrient, and energy metabolism (6). Gut microbiota dysbiosis is associated with the development of many diseases and metabolic disorders. It is reported that up to 95% dietary phytochemicals can't be absorbed in small intestine, but reach the large intestine and are metabolized by the gut microbiota (7). Hence, dietary polyphenols are important in ameliorating abnormal gut microbiota. Lu et al. investigated the effects of Anthocyanin-rich blue potato on gut microbiota composition and short-chain fatty acids (SCFA) production with computer-controlled batch culture fermentation system containing human fecal microbiota. The results revealed that short-term exposure to polychlorinated biphenyl (PCBs) led to decreased abundance and altered gut microbiota profiles as well as lowered SCFA and acetate levels. Anthocyanin-rich potatoes counteract PCB-mediated disruptions in human gut microbiota profiles and SCFA production.

Dendropanax morbifera (DM) is a medicinal plant rich in polyphenols. Awais et al. analyzed the polyphenol contents of DM from different regions and evaluated their anti-obesity effects using 3T3-L1 cell model. The anti-obesity effects of DM extracts were related to their polyphenol contents via down regulating adipogenic markers and upregulating thermogenic markers. Wu et al. investigated the changes of secondary metabolites and related metabolic pathways in different Jianghua Kucha (JHKC) tea tissues via metabolomics analysis, including the bud, 1st-4th leaves, and new stem. The results offer guidelines for efficiently utilizing specialized metabolites of JHKC in the future.

In summary, the results of the above-mentioned studies represent some new relevant data on the effects and mechanisms of phytochemicals on glycolipid metabolism. However, it is regret that there is no study about the absorption and metabolism of plant secondary metabolites in this Research Topic. Previously, we have investigated the absorption and metabolism of astilbin and its aglycon, taxifolin, in rat (8, 9). Besides, zein nanoparticles were used as the carrier to improve the bioavailability of many flavonoids, including astilbin (10), taxifolin (11), quercetin (12), dihydromyricetin (13), and luteolin (14). There are many strategies to improve the bioavailability of phytochemicals *in vivo*, such as liposomes, emulsions, particles, solid dispersions, cocrystals, etc. Lewandowska et al. have summarized these strategies in a review article (15). It is a regret that no *in vivo* human studies are included in this Research Topic. In addition to poor bioavailability, the absorbed phytochemicals also undergo phase I and phase II metabolism and produce many metabolites *in vivo* (3, 4). Whether these metabolites can also exhibit the activities or functions of native phytochemicals requires more research.

## Author contributions

Q-FZ: Conceptualization, Data curation, Writing – original draft, Writing – review & editing. W-JW: Writing – review & editing. SC: Writing – review & editing. G-DZ: Writing – review & editing.
